# Allopregnanolone Reinstates Tyrosine Hydroxylase Immunoreactive Neurons and Motor Performance in an MPTP-Lesioned Mouse Model of Parkinson's Disease

**DOI:** 10.1371/journal.pone.0050040

**Published:** 2012-11-29

**Authors:** Samuel O. Adeosun, Xu Hou, Yun Jiao, Baoying Zheng, Sherry Henry, Rosanne Hill, Zhi He, Amar Pani, Patrick Kyle, Xiaoming Ou, Thomas Mosley, Jerry M. Farley, Craig Stockmeier, Ian Paul, Steven Bigler, Roberta Diaz Brinton, Richard Smeyne, Jun Ming Wang

**Affiliations:** 1 Department of Pathology, University of Mississippi Medical Center, Jackson, Mississippi, United States of America; 2 Department of Psychiatry and Human Behavior, University of Mississippi Medical Center, Jackson, Mississippi, United States of America; 3 Department of Pharmacology and Toxicology, University of Mississippi Medical Center, Jackson, Mississippi, United States of America; 4 Program in Neuroscience, University of Mississippi Medical Center, Jackson, Mississippi, United States of America; 5 The Memory Impairment Neurodegenerative Dementia Research Center, University of Mississippi Medical Center, Jackson, Mississippi, United States of America; 6 Department of Developmental Neurobiology, St. Jude Children's Hospital, Memphis, Tennessee, United States of America; 7 Department of Pharmacology and Pharmaceutical Sciences, University of Southern California, Los Angeles, California, United States of America; Emory University, United States of America

## Abstract

Restorative/protective therapies to restore dopamine neurons in the substantia nigra pars compacta (SNpc) are greatly needed to effectively change the debilitating course of Parkinson's disease. In this study, we tested the therapeutic potential of a neurogenic neurosteroid, allopregnanolone, in the restoration of the components of the nigrostriatal pathway in MPTP-lesioned mice by measuring striatal dopamine levels, total and tyrosine hydroxylase immunoreactive neuron numbers and BrdU-positive cells in the SNpc. An acute treatment (once/week for two weeks) with allopregnanolone restored the number of tyrosine hydroxylase-positive and total cell numbers in the SNpc of MPTP-lesioned mice, even though this did not increase striatal dopamine. It was also noted that MPTP treated mice to which allopregnanolone was administered had an increase in BrdU-positive cells in the SNpc. The effects of allopregnanolone in MPTP-lesioned mice were more apparent in mice that underwent behavioral tests. Interestingly, mice treated with allopregnanolone after MPTP lesion were able to perform at levels similar to that of non-lesioned control mice in a rotarod test. These data demonstrate that allopregnanolone promotes the restoration of tyrosine hydroxylase immunoreactive neurons and total cells in the nigrostriatal tract, improves the motor performance in MPTP-treated mice, and may serve as a therapeutic strategy for Parkinson's disease.

## Introduction

Parkinson's disease (PD) affects nearly one million people in the US, with 50,000 new cases diagnosed annually. The symptoms of PD are closely associated with the depletion of striatal dopamine (DA) brought about by the degeneration and death of DAergic neurons in the midbrain substantia nigra pars compacta (SNpc). While replenishing DA with its precursor L-DOPA and deep brain stimulation into subcortical regions can provide temporary relief of the typical parkinsonian symptoms, there is a great need for restorative/protective therapies to effectively change the debilitating course of the disease.

We, and others, have hypothesized that promoting the proliferation of pre-existing *endogenous* neural stem/progenitor cells (NS/PC) in the brain may be a viable strategy to treat neurodegenerative diseases such as Alzheimer's disease (AD) and PD [1]–[5]. Our recent studies, as well as those of others, demonstrated that allopregnanolone (APα, 3α-hydroxy-5α-pregnan-20-one), a small molecule that freely penetrates the blood-brain barrier, is a proliferative factor for NS/PC in human, rat, and mouse [3], [6]–[9]. In a triple transgenic mouse model of AD (3×TgAD, (APPSwe, PS1M146V, tauP301L)), APα was shown *in vivo* to reverse neurogenic deficits in both of the recognized postnatal proliferative zones of the brain – the subventricular (SVZ) and subgranular zone (SGZ), to levels comparable to the normal non-transgenic age-matched control [2], [3]. APα also functionally restored learning and memory function in these same transgenic mice [2], [3], [10], [11]. More relevant to the current study is our recent work which demonstrated that APα reversed the loss of tyrosine hydroxylase (TH) expressing neurons in the SNpc of 3×TgAD mice, likely by promoting the generation of new TH-positive neurons [12].

In this study, we investigated the impact of APα on the loss of DA neurons in the SNpc, depletion of striatal dopamine, and deficits in motor performance; that are observed following acute administration of 1-methyl-4-phenyl-1,2,3,6-tetrahydropyridine (MPTP), a chemical toxin that produces parkinsonian pathology in mice [13].

## Results

### Effects of APα on levels of dopamine and norepinephrine in MPTP-lesioned C57BL/6 mice

**Figure 1:**
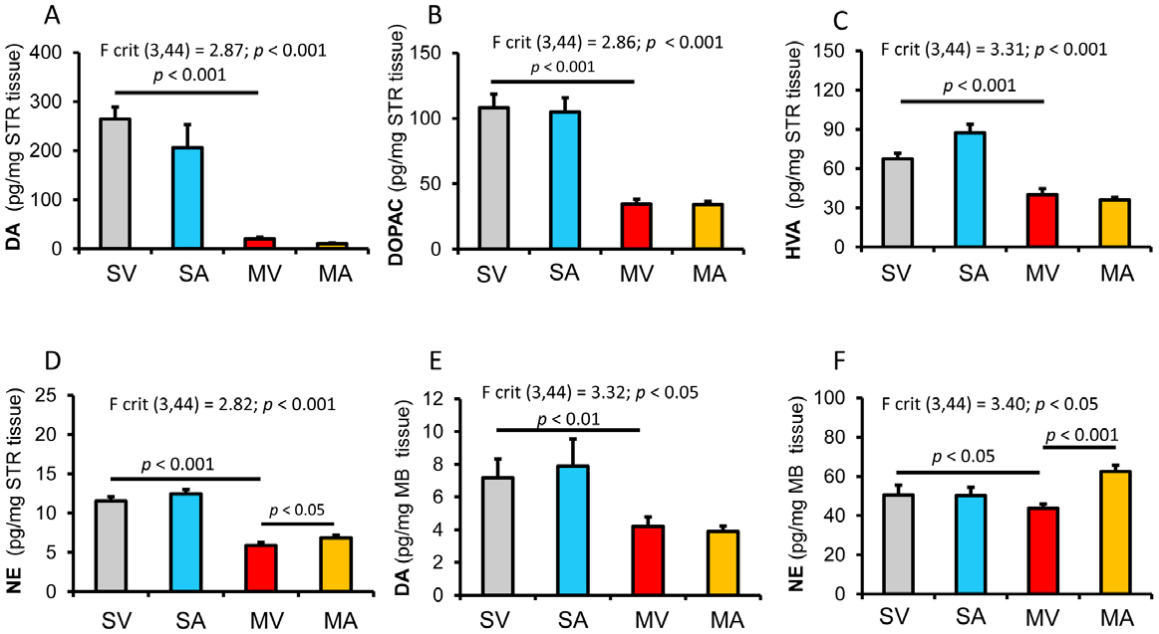
Effects of Allopregnanolone on Dopamine and Norepinephrine Levels. The concentrations of (**A**) dopamine (DA), (**B**) 3,4-dihydroxyphenylacetic acid (DOPAC), (**C**) homovanillic acid (HVA), and (**D**) norepinephrine (NE) in striatum (STR), and (**E**) DA and (**F**) NE in midbrain (MB) are presented as average ± SEM. The statistical significances were analyzed by Two-way ANOVA with replications followed by post-hoc t-test (two sample assuming equal variance). The data indicated that MPTP-lesion significantly reduced the DA concentration, as well as its metabolites, DOPAC and HVA, in both striatum and midbrain. APα did not affect the DA concentration in both striatum and midbrain. However, the MPTP-lesion induced reductions of NE levels were significantly reversed by APα in both striatum and midbrain. SV = saline+vehicle; SA = saline+APα; MV = MPTP+vehicle; and MA = MPTP+APα.

Twenty-four days after MPTP injection, we observed a 92% reduction of striatal DA, from 264.4±25 pg/mg wet tissue in non-lesioned C57BL/6 mice (SV) to 20.5±2.6 pg/mg wet tissue in MPTP-treated mice (MV, *p*≤0.001 vs. SV). We also noted a significant reduction of DA metabolites, 3,4-dihydroxyphenylacetic acid (DOPAC, 68%) and homovanillic acid (HVA, 40%) in the striatum. In addition to the DA loss, MPTP also induced a 48 percent decrease in the levels of another monoamine neurotransmitter norepinephrine (NE) (*p*≤0.001) in the striatum (Figure 1).

At day 17 following the first APα administration, we found no significant changes in either the total striatal DA levels, or striatal DA turnover (determined by measurements of DOPAC and HVA) when we compared SV to SA or MV to MA (Figure 1). We did, however, observe that APα treatment induced a small but significant increase in striatal NE in MPTP-treated mice compared to MPTP mice treated with vehicle (p≤0.03, MV vs. MA) (Figure 1). In the midbrain, APα also did not increase DA levels in control (SA) or MPTP-treated mice (MA), but it significantly augmented levels of NE in mice treated with MPTP (p≤0.001, MV vs. MA) (Figure 1).

### APα increases the expression of tyrosine hydroxylase protein in the midbrain of MPTP-lesioned mice

**Figure 2:**
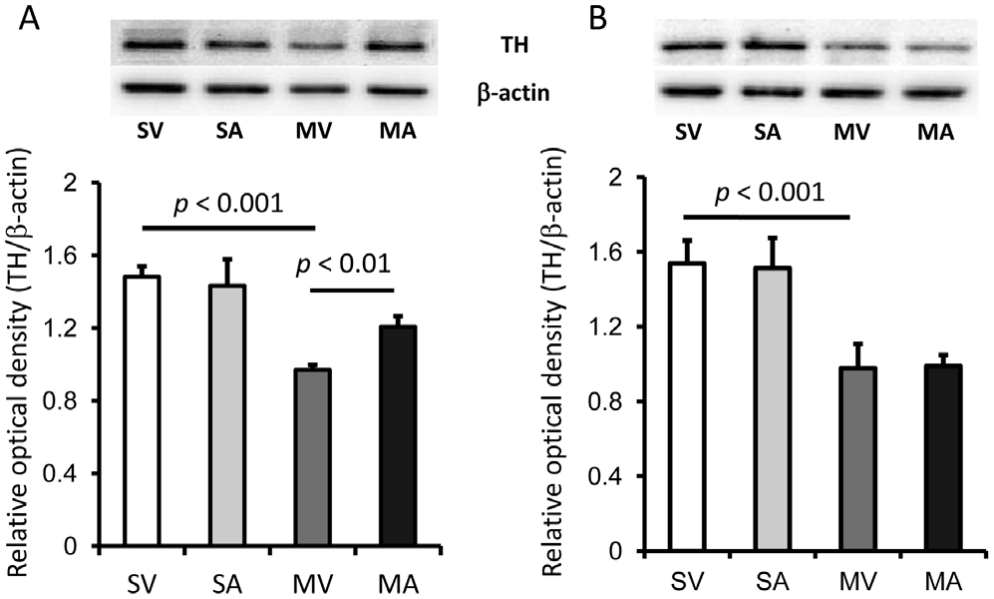
Allopregnanolone reverses the MPTP-induced decrease in tyrosine hydroxylase expression in the midbrain of mice that were tested on rotarod. The expression of TH in midbrain protein extracts was analyzed by Western blot in mice that underwent behavioral tests (**A**) and those did not (**B**). The relative optical density of TH was normalized by the internal loading control β-actin and presented as mean ± SEM (n = 7 per group in A, and 5–6 per group in B). SV stands for Saline+vehicle, SA stands for saline+APα, MV stands for MPTP+vehicle, and MA stands for MPTP+APα.

The expression of TH protein levels were measured by Western blot in midbrain extracts. The average level of TH-IR in the midbrain of MPTP-lesioned mice (MV) was decreased by 35% compared to non-lesioned C57BL/6 mice (SV, *p*≤0.04). In mice that were subjected to behavioral tests, TH-protein expression was about 25% higher in those treated with APα (MA) than that in the MPTP-lesioned mice that did not receive APα (MV, *p*<0.0, Figure 2A). In mice that had not been tested for motor performance, APα treatment had no effects on TH protein expression (Figure 2B). APα had no effects on TH-IR levels in non-lesioned C57BL/6 mice whether tested on motor performance or not (Figure 2).

### APα restores the number of tyrosine hydroxylase immunoreactive neurons in the SNpc of MPTP-lesioned mice

**Figure 3:**
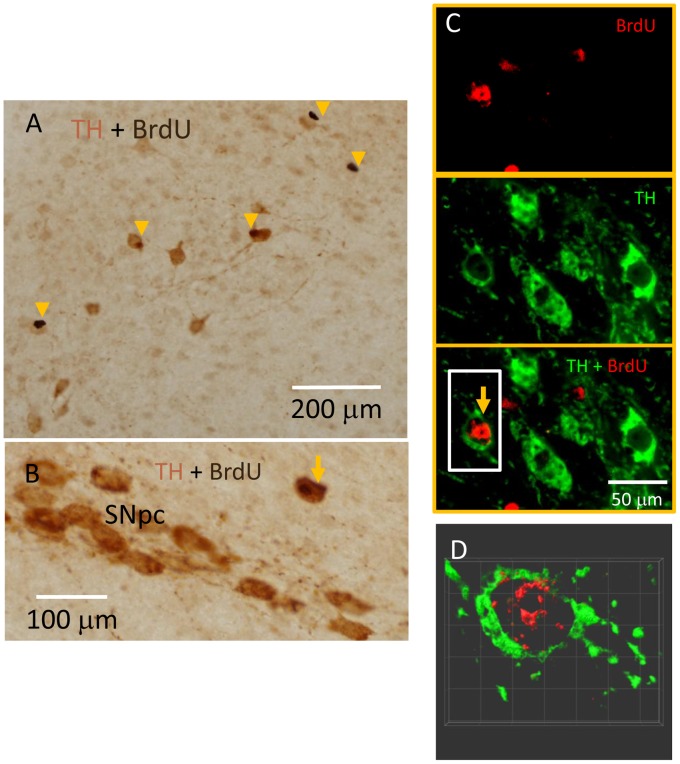
BrdU-IR and TH-IR double positive cells are readily observed in SNpc. Serial brain sections were double immunolabeled for BrdU and TH (prepared by NeuroScience Associates) using NiDNB/DAB color reaction(A and B). The BrdU-IR nuclei are dark coffee while the TH-IR in cytoplasm is light reddish brown. The double immunolabeling of BrdU and TH was further visualized by fluorophore labeling methods (C). The TH-IR is in cytoplasm (green, upper panel), BrdU-IR is in nucleus (red, middle panel). The merged image of TH and BrdU double staining is presented in the lower panel of C. The BrdU positive cells are indicated by arrowheads and BrdU-IR/TH-IR double positive cells are indicated by arrows. The 3D volume-view of z-series images of a BrdU-IR/TH-IR newly formed TH neuron (inside a white box in C) is presented in D and Figure S1. Figure 3D and S1 demonstrates a true co-localization of BrdU-IR (red, nucleus) and TH-IR (green, cytoplasm) in the same cell.

Treatment with the neurotoxin MPTP resulted in a loss of TH-IR neurons in SNpc, which parallels the DA neuron loss seen in PD [13]. An unbiased stereology approach was used to evaluate the effects of APα on the number of TH-IR neurons and the number of new cells labeled by BrdU, a thymidine analogue incorporated during DNA amplification in the S-phase of the cell cycle, in SNpc in mice 7 days after MPTP-lesion [2], [3]. Forty mouse brain hemispheres (ten per group) were embedded in one block and sectioned to obtain 40 µm free-floating serial sections from the rostral to caudal pole of the SN. Every 6th section (the first one was chosen randomly) was immunolabeled for BrdU and TH using a NiDAB/DAB color reaction for visualization of IR cells (prepared by NeuroScience Associates). The representative images are shown in Figure 3. The DAB color reaction immunohistochemistry was further confirmed by fluorophore labeling methods. In the sections developed by colorimetric methods, the BrdU-IR nuclei are dark brown, while the TH-IR cytoplasm of neurons are light brown (Figure 3A and 3B), The BrdU-IR cells are indicated by arrowheads, and the double positive (BrdU-IR/TH-IR) neurons are indicated by arrows. High magnification deconvoluted images using 1 micron z-stacks of immunofluorescent labeled sections confirmed that BrdU-IR (red) in the nuclei co-localized with TH-IR (green) in the cytoplasm (Figure 3C). The 3-dimensional volume view of the newly formed TH-positive neuron in Figure 3C (in a white box) is shown in Figure 3D and Figure S1.

**Figure 4:**
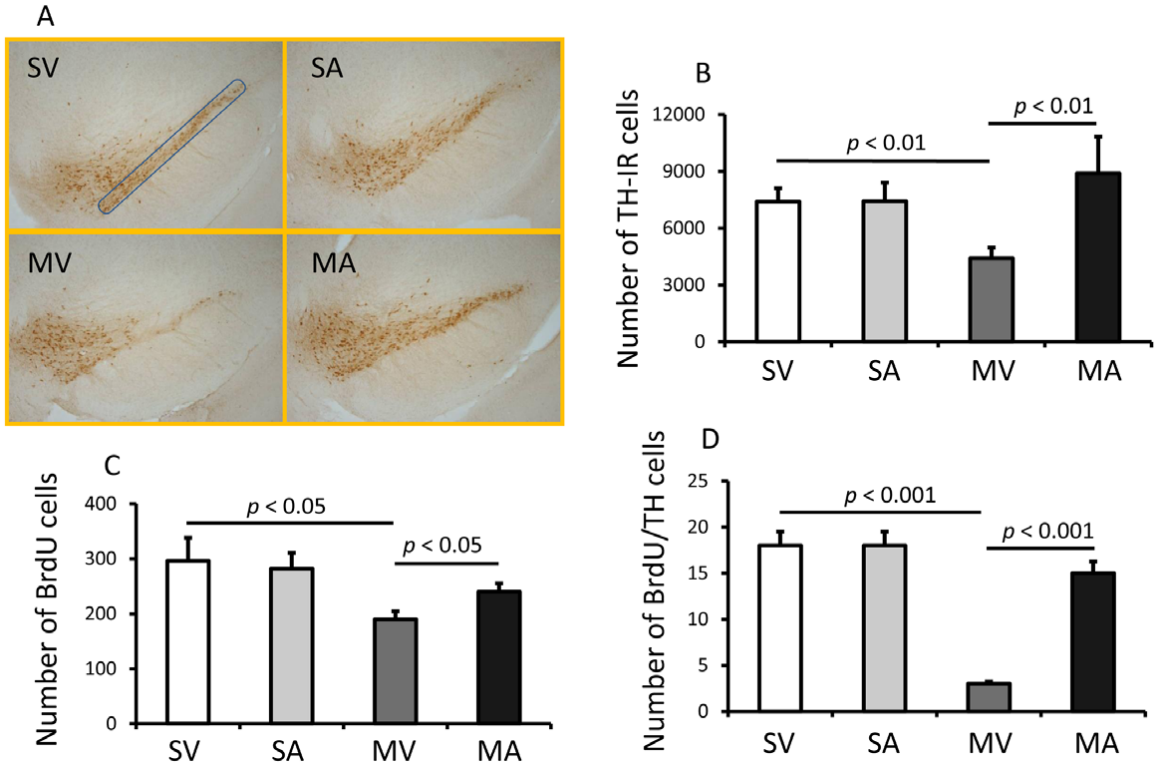
APα Increases the Number of TH-IR Cells in SNpc of MPTP-lesioned Mice. The numbers of the TH positive cells in the SNpc was counted on serial coronal sections using Slidebook stereology module. A. Representative images of SNpcs of mice receiving saline+vehicle (SV), saline+APα (SA), MPTP+vehicle (MV), or MPTP+APα (MA). B. The number of TH-IR cells. C. The number of BrdU-IR cells. D. The number of BrdU-IR/TH-IR cells. The results are presented as mean ± SEM. SV stands for Saline+vehicle.

The number of TH-neurons in SNpc was estimated using the unbiased stereology module in SlideBook 5.0 [2], [3] (Figure 4A). The estimation indicated there were 7424±992 (the coefficient of error (CE) = 375) TH-IR neurons in the saline plus APα group (SA) and 7401±711 (CE = 269) in the saline without APα group (SV). This demonstrates that APα application did not affect TH-IR neuron number in normal controls (Figure 4B). The number of TH-IR neurons in SNpc in mice lesioned with MPTP (MV) was estimated at 4416±565 (CE = 213), indicating that MPTP-lesion induced a loss of approximately 40% of TH-IR neurons compared to both the SV and SA treated mice (p≤0.01). Following APα (MA) treatment, mice lesioned with MPTP showed no SNpc TH-IR neuron loss (8896±1945, CE = 735, p≤0.01 vs. MV, Figure 4B). Since APα was administered 7 days after MPTP, a time when neuron loss is well established [14], [15], these results suggest that APα restored the number of TH-IR neurons in the SNpc of mice treated with MPTP to similar levels as in normal mice.

### APα treatment induces the generation of new neurons in the substantia nigra pas compacta of MPTP-lesioned mice

To determine the potential mechanism underlying the recovery of the number of TH-IR neurons in SNpc, we analyzed the BrdU-IR cells in the SNpc. The BrdU-IR cells were 282±42 (CE = 16) in SA, and 296±28 (CE = 11) in SV mice. In mice treated with MPTP (MV), the number of BrdU-IR cells was reduced by 33% to 190±15 (CE = 6) vs. SA (p≤0.04), and reduced by 36% vs. SV (p≤0.04). In MPTP-lesioned mice that received APα, we observed a significant increase in the number of BrdU-IR cells in the SNpc to 240±15 (CE = 6, p≤0.04 vs. MV), and this value was not significantly different from either SA or SV (Figure 4C).

To determine how many BrdU-positive cells were also TH-IR neurons, double-labeled BrdU-IR/TH-IR neurons in the SNpc were counted. We found that the MPTP-lesion resulted in a six-fold decrease in BrdU-IR/TH-IR neurons from 18±1.2 in the SV group to 3±1 in MV group (p≤0.001). When treated with APα 7 days after MPTP, the number of BrdU-IR/TH-IR neurons increased to 15±3 (p≤0.001 vs. MV), a five-fold increase compared to the MV group (Figure 4D).

### APα restores the numbers of Nissl-stained cells and neurons in the SNpc of MPTP-lesioned mice

**Figure 5:**
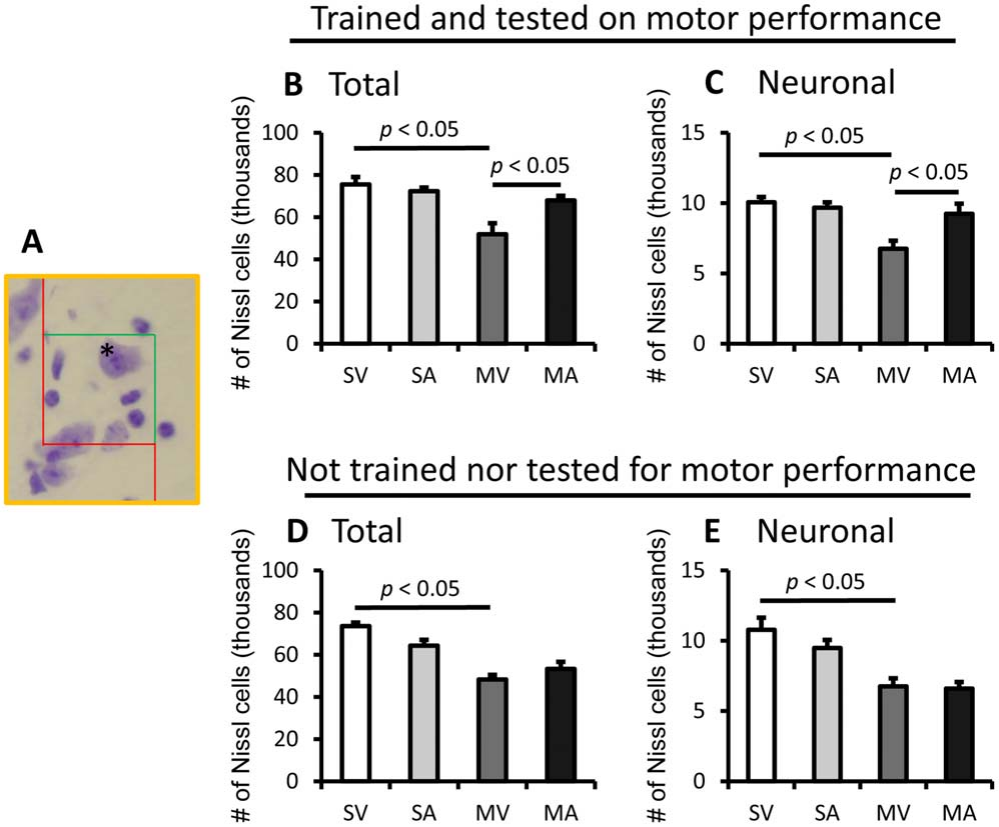
APα Restores the Number of Total Cells and Neurons in SNpc of MPTP-lesioned Mice. The numbers of the Nissl stained total cells and neurons were counted using Slidebook stereology module. **A**. A Representative image of an optical fractionator, * indicates a countable Nissl neuron which is within the counting frame. **B** and **D**. The numbers of total (neuronal and non-neuronal) Nissl stained cells in SNpc of mice. **C** and **E**. The number of Nissl stained neuronal cells. The results are presented as mean ± SEM. SV stands for Saline+vehicle, SA stands for saline+APα, MV stands for MPTP+vehicle, and MA stands for or MPTP+APα.

To determine whether the APα effect was specific to the SNpc DA neurons, we used unbiased stereology to estimate both total number of Nissl-stained cells and total neurons in the SNpc of MPTP-lesioned mice (Figure 5). Neurons were distinguished from non-neuronal cells on the basis of size, the presence of euchromatin in the nucleus and surrounding cytoplasm, and a clearly visible nucleolus [16]. In mice tested for motor behavior, the total number of Nissl-stained cells in the SNpc was estimated to be 75536±7030 (CE = 2661), and the total number of Nissl-stained neurons was 10064±741 (CE = 281), in untreated mice (SV). These values are consistent with the reported numbers in C57BL/6 mouse SNpc [12], [17]. APα showed no effects on the number of total Nissl-cells (72304±3535, CE = 1338) nor on the number of Nissl-neurons (9680±749, CE = 284). In MPTP-lesioned mice (MV), the total number of Nissl cells showed a 28% decrease to 53894±8143 (CE = 3094) and a 33% decrease of Nissl-stained neurons to 6752±1147 (CE = 436) in comparison with SV mice. Treatment with APα (MA) reversed the reduction of Nissl-stained total cells (67944±6662, CE = 2531) and Nissl-stained neurons (9240±1435, CE = 545), revealing a 26% and a 37% increase, respectively, when compared to MV mice (Figure 5B–C). In mice that did not undergo motor performance tests, the numbers of Nissl stained total cells (73608±3331, CE = 1265, for SV and 62832±3175, CE = 1206, for SA) and neurons (10776±1716, CE = 669, for SV and 9480±1142, CE = 433, for SA) in saline control and MPTP-lesioned (48288±4391, CE = 1669, and 6432±1267, CE = 481, for MV) mice were similar to those of the mice trained and tested for motor performance. However, no significant effect of APα treatment was observed (53312±6661, CE = 2531, for MA, Figure 5D–E). Thus, the APα treatment appears not only to increase the number of the DA neurons, but also other cells that are located in the SNpc of MPTP-lesioned mice that were forced to run on the rotating rod during the behavioral tests.

### APα reverses balance and coordination deficits of MPTP-lesioned mice in the rotarod test

**Figure 6:**
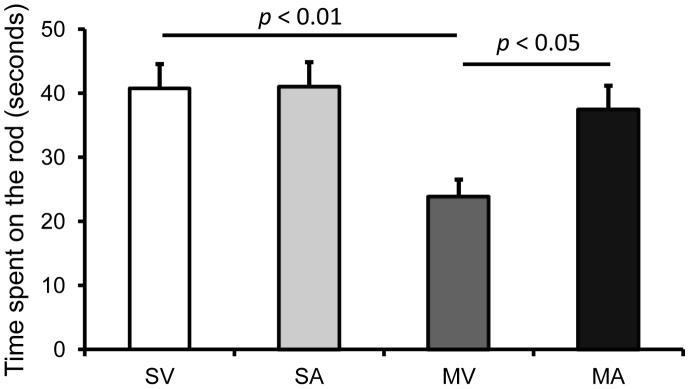
APα Improves the Rotarod Performance of MPTP-lesioned Mice. The mice were tested for Rotarod performance that measures the riding time (seconds) or endurance, indicating the balance and coordination performance of the subjects. The time of mice staying on the rod was plotted as mean ± SEM. The data were analyzed using ANOVA (F(3, 44) = 5.10, p = 0.0026). SV stands for Saline+vehicle, SA stands for saline+APα, MV stands for MPTP+vehicle, and MA stands for or MPTP+APα.

MPTP-lesions significantly decreased performance on the rotarod to 58% of the saline-treated C57BL/6 control mice from 40.75±3.80 sec (SV) to 23.75±2.75 sec (MV) (*p*<0.01) (Figure 6). APα had no significant effect on rotarod performance in saline treated control mice (SA, 40.88±3.96 sec, *p* = 0.49 vs. SV). However, APα significantly reversed the reduction in the time of staying on the rod elicited by MPTP lesion to 37.42±3.74 sec (MA, *p*<0.05 vs. MV).

### APα increases the number of TH-IR cells in LC of MPTP-lesioned mice

**Figure 7:**
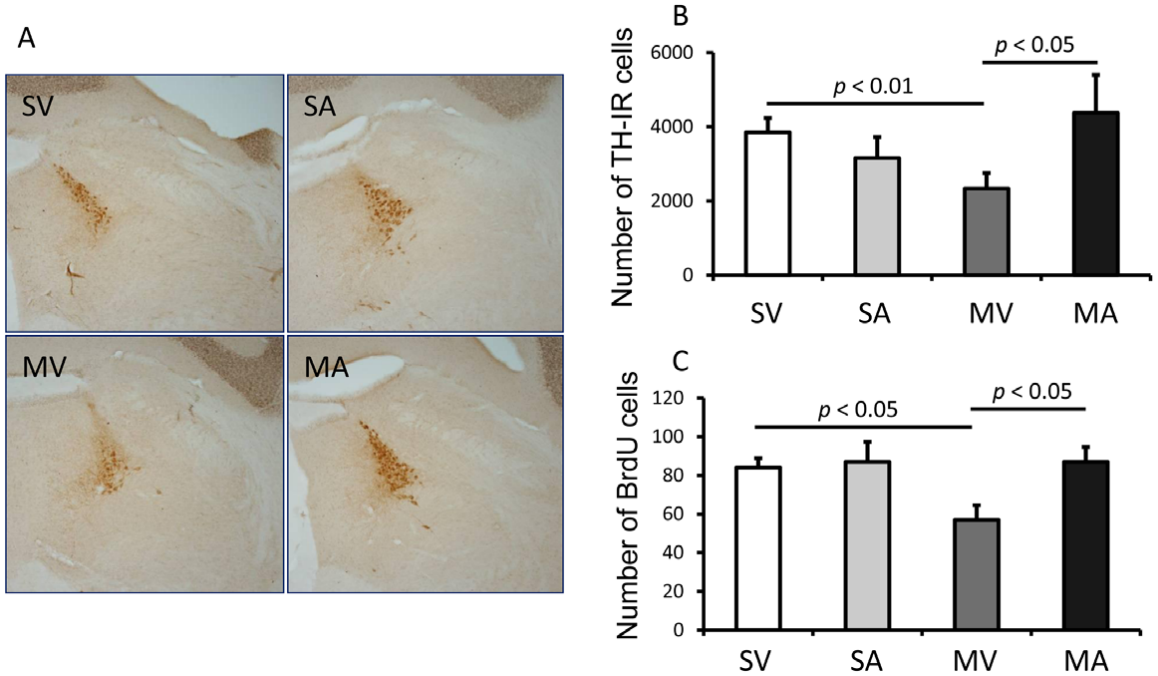
APα restores the number of TH-IR cells in locus coeruleus of MPTP-lesioned mice. Locus coeruleus is a brain structure where the majority of the neurons containing NE. **A**. Representative images of TH-IR cells in locus coeruleus. **B**. The numbers of TH-IR cells. **C**. The number of BrdU-IR cells. SV stands for Saline+vehicle, SA stands for saline+APα, MV stands for MPTP+vehicle, and MA stands for or MPTP+APα.

As demonstrated above, APα treatment (once/week for two weeks) significantly reversed the MPTP lesion-induced effects on 1) motor performance, 2) the number of TH-expressing neurons in the SN, 3) the expression of TH protein, and 4) the levels of NE in the SN and midbrain. APα treatment, however, did not reverse the MPTP-lesion induced decrease of DA levels in both striatum and midbrain. These data suggest that the increased number of TH-IR cells, which either come from the recovery of dying TH-neurons or as we hypothesize, from newly generated neurons, may preferentially synthesize NE. To examine this hypothesis, we estimated the number of TH-IR neurons in the LC, an area rich in NE neurons, to determine whether MPTP and/or APα altered the number of NE synthesizing-neurons (Figure 7). MPTP-lesion resulted in a 40% decrease in the number of TH-IR neurons in the LC (SV, 3856±376 vs. MV, 2336±421, *p*≤0.01). Treatment with APα reversed the effect of the MPTP-lesion in regard to the number of LC TH-IR neurons (MV, 2336±421 vs. MA,4378±1023, p≤0.01) (Figure 7B). MPTP administration also resulted in a 33% reduction in the number of BrdU-IR cells in LC (SV, 84±5 vs. MV, 57±8, p = 0.024). In MPTP-lesioned mice that received APα, there was a 53% increase in BrdU-IR cells in the LC (87±8 in MA) compared to the MPTP-lesioned mice that received vehicle (MV) (Figure 7C).

## Discussion

In this study, we demonstrate that APα treatment (once a week for two weeks) improved motor performance and restored TH expression, the number TH-IR neurons and the total cells in SN following MPTP-lesion. These results suggest that APα has potential to function as a therapeutic agent for PD by restoring the components of the nigrostriatal pathway. This conclusion is supported by the fact that APα treatment was not started until 1 week after completion of the MPTP administration, a time when the lesion induced by acute administration of MPTP-induced lesion is established [14].

### APα improves the balance and coordination of MPTP-lesioned mice

The balance and coordination of mice were tested in rotarod performance task using a rotating rod where mice are forced to engage in motor activity to prevent them from falling. In the current study, mice treated with APα demonstrated an almost completely reversal of the deficits in balance and coordination that were induced by MPTP in C57BL/6 mice [18]. This suggests that peripheral administration of APα can facilitate functional restoration of motor performance, particularly in the modalities of balance and coordination. A previous report indicated that exercise was able to improve the motor function of MPTP-lesioned mice without any effects on striatal DA levels [19]. This apparent DA-independent improvement in motor function is similar to our findings that APα treatment improves motor performance without any significant increase in striatal DA, DOPAC or HVA levels.

Unlike the lack of effects on striatal dopamine, we did observe that APα treatment significantly reversed the MPTP-induced decrease in striatal and midbrain NE. The increase in NE levels might be sufficient to explain the beneficial behavioral effect of APα, based on numerous reports on the role of the noradrenergic system in the pathology of PD. NE and DA share a common biosynthetic pathway. Additionally, stimulation of the LC enhances burst firing in the SNpc DA neurons, and such bursts are reduced by prazosin, an α1 noradrenergic receptor antagonist [20], [21]. The neuroprotective effects of NE are also seen in human tissue where brains from PD patients that had higher levels of NE showed a lower degree of SNpc DA neuron loss [22]. Moreover, in PD brains, the loss or degeneration of DA neurons may actually be preceded by the loss of NE neurons in the locus coeruleus and the loss of NE neurons may surpass the loss of DA neurons [23]–[28]. Thus, NE or NE neurons may be as important as DA or DA neurons in PD.

The increased generation of LC neurons with a subsequent increase in NE in MPTP-lesioned mice treated with APα that we observed, supports a mechanism where alterations in the NE system may precede the restoration of DA neurons in the SNpc as well as dopamine levels in the striatum. As discussed above, increase in NE and NE neurons may be in and of themselves beneficial to motor improvement.

### New tyrosine hydroxylase cells in SNpc

The increased TH-IR in APα treated MPTP-lesioned mice may be from the recovery of degenerating TH neurons by MPTP-lesion and/or newly generated (or differentiated) TH expressing neurons. Our stereological assessment of both TH-IR and Nissl stained cells demonstrates that MPTP not only reduced the number of TH-IR neurons in SNpc, but also the number of total cells, including other non-DA neurons and glial cells. These results suggest that new cells were added into the SNpc of the MPTP-lesioned mice, although the sources or the portion of these new cells have not been determined and they are either 16 days or 9 days of age given our BrDU injection regimen.

Interestingly, the neurorestorative effects of APα were only observed in mice that were subjected to motor performance tests; hence the effects of APα may need to be enhanced or maintained with some forms of physical activities, which in this case was the two days rotarod performance. In these two days, mice were forced to engage in motor activity to prevent them from falling. A question may be raised as to how only two days of forced running on a rod can produce similar restoration effects on TH expression and TH-IR neurons in SN as reported recently by utilizing running wheels or forced treadmill for several weeks [29], [30]. In the current study, it should be noted that the two days rotarod performance followed treatment with a neurogenic agent, APα, administered once a week for two weeks. Therefore, we speculate that the two days forced physical activity helps the newly formed cells induced by APα to survive and differentiate into TH expressing neurons. Our hypothesis is supported by the fact that newly formed neural progenitors can differentiate into TH-expressing neurons within 24 hrs when exposed to basic fibroblast growth factor (bFGF2) and glial cell conditioned media [31]. Interestingly, it has been reported that physical exercise increases bFGF2 expression [16].

The SNpc is not recognized as a classical neurogenic niche. However, a number of studies have reported that MPTP [32]–[35], 6-OHDA [36], and even treatment with a dopamine receptor antagonist [37], [38], can lead to the generation and differentiation of new SNpc DA neurons. In the present study, we demonstrated that APα restored the numbers of total cells (including TH-IR and other Nissl-positive cells) in the SNpc of MPTP-lesioned mice.

We have no evidence so far to support the likelihood that these APα-restored TH-positive neurons are generated locally within the SNpc, neither can we exclude the possibility that these cells have migrated from a known neurogenic region such as the SVZ, or whether they recovered from the dying cells after MPTP-lesion. It is possible that APα promotes the proliferation of glial cells in SNpc, and that these cells can further promote the formation or differentiation of new TH neurons or themselves differentiate into newly formed TH-positive neurons. This hypothesis is supported by our data showing that APα increased the number of BrdU-IR cells, TH-IR neurons and double-positive neurons, in SNpc of MPTP-lesioned mice. In addition, the ratios of double positive cells versus BrdU-IR cells in SNpc in non-lesioned mice (18/282) and APα-treated MPTP-lesioned mice (15/240) are 6.4% and 6.3% respectively, while this ratio in MPTP-lesioned mice is only (3/190) 1.5%. APα appears to also reverse the deficits in differentiation of newly formed cells into TH positive neurons in SNpc of MPTP-lesioned mice.

Accumulated evidence now suggests that there are additional neurogenerative niches in the brain apart from the hippocampal dentate gyrus SGZ and the cerebral SVZ. These include the hypothalamus [39], cerebellum [9], [40]–[45], and substantia nigra [32], [34], [35], [46]–[48]. Additionally, recent studies have demonstrated that the primary progenitors in adult neurogenesis are astrocyte-like cells that express glial fibrillary acidic protein (GFAP) and that surviving cells exhibit neurites seven days after proliferation [49], [50]. In support of this hypothesis, GFAP-expressing mesencephalic progenitor cells can differentiate into TH-IR neurons within 4 days by sonic hedgehog, a key protein regulating organogenesis of the vertebrate brain [51]. Furthermore, the BrdU labeled adult subependyma cells of the lateral ventricle differentiate into TH-expressing neurons after 24 hr exposure to bFGF2 and glial cell conditioned media [31]. Therefore, it is possible that in the SNpc, as well as cerebellum and other brain areas, a subgroup of glial-like cells are proliferating and generating new cells that have the capacity to differentiate into both neurons and glial cells as regulated by their microenvironment.

### APα is a neurogenic agent

APα is synthesized throughout the embryonic period in pluripotent progenitor cells [52], [53] as well as in neurons [54], [55]. The highest concentration of APα, 20–30 times higher than any other time in life, occurs in late gestation [56] when most of the CNS neurons are generated and functional structures are formed. These data suggest a neurogenic function for APα.

In contrast to the developing brain, the concentration of APα is significantly reduced in the brains of humans with AD [57], [58] as well as those from a transgenic mouse model of AD [2], [3]. In Parkinson's disease patients, the levels of APα are lower in both the cerebrospinal fluid and plasma, and the synthesis of APα is reduced in the SN and caudate nucleus compared with age-matched controls [59], [60]. The severity of each of these neurodegenerative diseases and the pathology appears to be inversely correlated with the levels of APα [58].

Recently, several reports have demonstrated that administering progesterone, the precursor of APα, during the first few hours to days after injury significantly limits central nervous system damage, reduces the loss of neural tissue, and improves functional recovery [61], [62]. APα was found to be more efficacious than progesterone when administered after injury [63]. In our previous study, we demonstrated that both progesterone and APα promoted *in vitro* proliferation of neural progenitor cells and that APα had greater efficacy than a similar concentration of progesterone [6]. These data suggest that APα a progesterone metabolite, is a primary effective agent for neuroprotection and also for neurogenesis.

The current work, demonstrating that APα reverses the decline in the number of TH-expressing cells in both SNpc and LC and the expression of TH protein in the midbrain of MPTP-lesioned mice, supports the neurogenic property of APα that was first reported in a mouse model of AD. The fact that at APα increased the proliferation of cerebellar neurogenic cells supports our current observation that APα is not only a neurogenic agent in known neurogenic areas such as SGZ, SVZ, but also in brain regions such as cerebellum [9] and SNpc [12]. These data suggest that APα is a potential therapeutic agent that may reduce or reverse symptoms of Parkinson's disease by inducing restoration of, and facilitating the incorporation of new, *endogenously-derived* DA neurons in the SNpc.

## Materials and Methods

### Animal and MPTP lesion

All mice used in this study were male C57BL/6J mice (Jackson Laboratory, Bar Harbor, Maine) that were 9 weeks of age at the beginning of the experiment. All mice were maintained in a temperature-controlled environment with free access to food and water and kept on a 12-h light/dark cycle from 7 am to 7 pm each day. All animal procedures were in compliance with University Mississippi Medical Center and St. Jude Children's Hospital and University Institutional guidelines and were approved by the UMMC (protocol # 1242) and SJCRH Institutional (protocol # 270) Animal Care and Use Committees.

**Figure 8:**
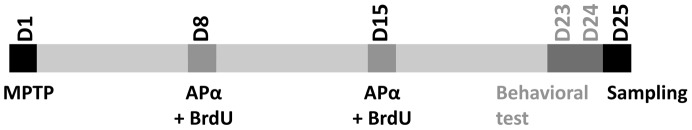
Experimental Design. Nine weeks old C57BL/6 male mice were randomly divided into 4 groups (SV, saline+vehicle; SA, saline+APα; MV, MPTP+vehicle; MA, MPTP+APα; 12–13/group). The mice were injected with MPTP (20 mg/kg)/2 h×4) or saline on day 1 (D1). The first APα (10 mg/kg of BW) and BrdU (100 mg/kg of body weight) injections were performed on day 8 (D8) and the second APα and BrdU injections were done on day 15 (D15) after MPTP injection. Seven mice of each group underwent behavioral tests on day 23 (D23) and day 24 (D24). The brain samples were collected on day 25 (D25) after MPTP injection for immunohistochemical and biochemical analyses.

A total of 50 mice were used, of which 24 were treated with saline and 26 mice were treated with the acute MPTP paradigm (20 mg/kg every 2 h×4 times) at SJCRH and were then transported in an air-conditioned vehicle to UMMC on the 4^th^ day after MPTP injection. None of the animals died during this transport. The influences of transportation-induced stress was controlled for by both saline and vehicle groups. The saline group and MPTP group were randomly divided into two groups. A total of 4 subgroups were generated: 1) Saline+Vehicle (SV, 12 mice), 2) MPTP+vehicle (MV, 13 mice), 3) saline+APα (SA, 12 mice), and 4) MPTP+APα (MA, 13 mice). On day 7 after MPTP injection, mice were subcutaneously injected with either vehicle (sterilized PBS containing 0.002% Ethanol) or APα (10 mg/kg in sterilized PBS containing 0.002% Ethanol). One hour after APα injection, all mice were given intraperitoneal BrdU (100 mg/kg). On day 15 after the MPTP-injection, the mice were injected with APα and BrdU again as was done on day 8. We have previously tested the dose-response relationship of APα [2], [3], [6], [10] with neurogenesis in SGZ and the treatment paradigms of 1/month single injection, 3/week for 3 months and once per week for 6 months [3], [64], [65]. The results indicated that the most effective regimen of APα is once per week at 10 mg/kg and BrdU applied one hour after APα treatment at 100 mg/kg labels the neurogenic effects of APα [3], [64], [65]. In the current work, APα treatment was initiated 7 days after completion of the MPTP administration, a time when the lesion induced by acute administration of MPTP-induced lesion is complete [14]. A second APα and BrdU treatment was given to increase the effects of APα and the number of BrdU labeled new cells in SN. One week after the second APα injection, 7 mice of each group were evaluated for motor performance once a day for two days (day 23 and 24). The remaining mice in each group were used to contrast the contribution of behavioral tests on neuropathology changes. On day 25, all mice were sacrificed and brain samples were collected (Figure 8).

### Tissue collection and preparation

Before sacrifice, the mice were anesthetized with 100 mg/kg ketamine and 10 mg/kg xylazine. After vascular flushing by cardiac perfusion with PBS, the brains were removed and dissected into two hemispheres. One hemisphere was fixed immediately in cold 4% paraformaldehyde for immunohistochemistry and unbiased stereological analysis. The striatum and midbrain were collected from the remaining fresh brain hemisphere in order to make extracts that can be used for measurements of protein, mRNA and monoamine levels from the same brain samples using our newly developed protocol.

This newly developed protocol reduces both cross subject variations and also reduces the number of animals/tissues needed for experiments. Briefly, the brain hemispheres were placed on an ice-cooled plate for dissection of the striatum and midbrain. The tissue was weighed and homogenized in RNase free water (10 µL/mg tissue) for 1 minute with a Bullet Blender (NextAdvance, Averill Part, NY) that can homogenize 24 samples simultaneously using 0.1 mm RNAse-free glass beads (NextAdvance, Averill Part, NY). 100 µl of each homogenate was transferred to a new ice-cold vial containing perchloric acid to reach a final concentration of 0.1 M for catecholamine measurement by HPLC. The tissue homogenates were centrifuged for 15 mins at a speed of 12,500 rpm at 4°C. The supernatant was passed through a 0.2 µm filter and stored at −70°C. The results obtained are similar with other reports using the traditional method [29], [66]. Protein and RNA were then extracted from the remaining two homogenate aliquots.

### Neurochemical analysis of dopamine, and metabolites

Quantification of dopamine and its metabolites were done on the perchloric acid extracts, using high pressure liquid chromatography with electrochemical detection (HPLC–ED) as previously described [29]. The signal from the electrochemical detector was recorded with an electronic data station (model SS420x, Scientific Software, Inc.). The amount of each individual chemical was determined by comparing the areas and heights of sample peaks with those from serial dilutions of the standard curve. Concentration of dopamine and its metabolites were expressed in pg/mg tissue weight.

### Immunohistochemistry using DAB as substrate for color development

Before immunolabeling, all slides were coded and the codes were not broken until analyses were completed. BrdU and TH double histolabelling (DAB/Ni color reaction) was performed by NeuroScience Associates and labeling conducted in every sixth section in the series as described previously (Wang et al., 2010). The BrdU-IR cells are dark coffee color and the TH-IR cells are light brown in the data presented.

### Immunofluorescence for TH and BrdU

Briefly, free-floating sections were incubated with rabbit anti-TH polyclonal antibody (1∶500 dilution, Pel-FreeZ® Analysis Certificate, Rogers, Arkansas), followed by incubation with Texas red (or FITC as the combination required) conjugated goat anti-rabbit IgG secondary antibody (1∶5000, Vector Laboratories, Burlingame, CA). After washing three times (5 min each) with PBST, the sections were fixed with 4% paraformaldehyde in PBS for 10 min. The sections were then denatured using 50% formamide in 2×SSC (NaCl 17.53 g, sodium citrate 8.82 g, pH 7.0) for 90 min at 65°C, 2N HCl solution for 30 min at 37°C, and then neutralized with sodium borate buffer (0.1 M, pH 8.5) for 2×5 min. After extensive washes with 0.01 M PBST (pH 7.4), the sections were incubated in blocking solution containing 0.1% Triton X-100 and 4% normal horse serum for 90 min. Subsequently, the sections were placed in an incubating solution containing 1% BSA with mouse anti-BrdU monoclonal antibody (1∶500 dilution, Novus Biologicals®, LLC, Littleton, CO) for 24 h at 4°C with gentle agitation, followed by fluorescein (or Texas red)-conjugated horse anti-mouse IgG secondary antibody (1∶5000 dilution, Vector Laboratories, Burlingame, CA). Immunoreactive controls were carried out by stepwise omission of antibodies or by replacement with normal serum. Immunoreactivity was visualized with Zeiss Axiovert 200 M fluorescent microscope as part of the 3iMarianas digital microscopy and a 63× SPlan apochromat oil objective (1.4 numerical aperture).

### Western Blot

Protein aliquots from the brain tissue homogenates were mixed 1∶1 with RIPA buffer mix (Sodium orthovanadate 1%, Protease inhibitor 0.1% and PMSF 1% in RIPA buffer). Protein concentration was determined by the BCA method before being prepared for gel electrophoresis. The protein extracts were prepared for gel electrophoresis by mixing 3 part protein with 1 part 4× Laemmli buffer. The protein-laemmli mix was heated with mixing at 95 degrees for 5 minutes and then allowed to cool on ice for 1 minute. Samples (30 µg each) were loaded on a 10% polyacrylamide gel and was run at 90 V for 3 hours. The protein was blotted on a PVDF membrane with affinity-purified rabbit anti-tyrosine hydroxylase antibody (Pel-Freez, Rogers, Arkansas US), at a concentration of 1∶2000. The antibody detected a protein of approximately 60 kDA in size. Protein sample loading was normalized with β-Actin which was detected on the same membrane after stripping. β-Actin antibody was used at a concentration 1∶10,000. The Pierce Fast Western Blot Kit, ECL substrate (Pierce Biotechnology, Rockfod Illinois, US) was used according to manufacturer instructions to detect the antibodies. The chemiluminiscent signal was detected using Bio-Rad ChemiDoc™ XRS+ system using optimal exposure times. Data are presented as relative optical densities of the individual bands (TH/β-Actin optical densities) ± SEM.

### Unbiased Stereology

The number of TH-labeled cells was determined in every sixth section in a series of 40 µm coronal sections using unbiased stereology (optical dissector) in the stereology module of SlideBook 5.0. The first section of each hemisphere was randomly started at the beginning of olfactory, and serial sections were collected to the end of the cerebellum. Systematic samplings of an unbiased counting frame of 50×50 µm^2^ within a 200×200 µm^2^ matrix spacing were produced using a semiautomatic stereology system (Zeiss Axiovert 200 M fluorescent microscope as part of the 3iMarianas digital microscopy. Positive cells that intersected the uppermost focal (exclusion) plane and those that intersected the exclusion boundaries of the unbiased sampling frame were excluded from analysis. Cells that met analysis criteria through a 20-µm z-axial distance were counted according to the optical dissector principle. The total positive cell number was multiplied by the virtual counted number with the reference factors (1/6 sections analyzed; 1/16 counting area; and the tissue shrink factor).

### Rotarod Performance

Animals were tested at different progressively higher speeds on the Rotarod apparatus that measures the ability of the animal to balance itself and remain on a rotating rod according to previously described methods [67], [68]. The total time that the animal was able to remain on the rod without falling off was recorded. When an animal fell off within the first 10 seconds of the test, the animal was re-tested after a short resting period. In all of the tests, the rod was cleaned with 70% ethanol before another subject was tested. No repeated training was done prior to testing but on day 1, animals were acclimatized to the equipment using the low speed of 7 rpm for a maximum time of 120 seconds. After this acclimatization trial, they were tested once at 10 rpm and allowed to balance on the working rotarod for up to 60 seconds [69]. On day 2, the animals were tested once each at speeds of 10 rpm, 13 rpm and 16 rpm with 60 seconds inter-trial interval. The average time spent on the Rod (latency) over the 4 testing episodes was analyzed for the four treatment groups.

### Statistical analysis

The statistical significances of the data were assessed by two-way ANOVA and a subsequent post hoc Tukey HSD test. A probability (p) value of 0.05 or less was considered statistically significant.

## Supporting Information

Movie S1 **The 3D volume-view of z-series images of a BrdU-IR/TH-IR newly formed TH neuron in SNpc (inside a white box in** **Figure 3C** **).** This 3D volume-view demonstrates a true co-localization of BrdU-IR (red, nucleus) and TH-IR (green, cytoplasm) in the same cell.
(MOV)
